# Cooperative progression of colitis and leukemia modulated by clonal hematopoiesis via PTX3/IL-1β pro-inflammatory signaling^☆^

**DOI:** 10.1016/j.gendis.2024.101397

**Published:** 2024-08-23

**Authors:** Hang He, Yuchen Wen, Qingran Huo, Hanzhi Yu, Jingjing Liu, Wenyan Jin, Zhiqin Wang, Huaquan Wang, Zhigang Zhao, Zhigang Cai

**Affiliations:** a National Key Laboratory of Experimental Hematology, Tianjin 300070, China; b Tianjin Key Laboratory of Inflammatory Biology, Tianjin 300070, China; c The Province and Ministry Co-sponsored Collaborative Innovation Center for Medical Epigenetics, Department of Pharmacology, School of Basic Medical Science, Tianjin Medical University, Tianjin 300070, China; d Department of Hematology, Tianjin Medical University Tianjin General Hospital, Tianjin 300070, China; e Department of Medical Oncology, Tianjin First Central Hospital, School of Medicine, Nankai University, Tianjin 300070, China; f Department of Rheumatology and Immunology, Tianjin Medical University Tianjin General Hospital, Tianjin 300070, China

Clonal hematopoiesis (CH) is a phenomenon in which hematopoietic stem cells carry genetic mutations with advantageous growth potential, resulting in aberrant expansion of immature and mature hematopoietic cell populations over time.^1^^,^^2^ Loss-of-function heterozygous mutations in *TET2* (tet methylcytosine dioxygenase 2) are among the most prevalent and significant drivers of CH and myelodysplastic syndromes, an age-related hematological disease.^3^ In addition, certain diseases, or environmental conditions, for example, atherosclerosis drive the onset and trajectory of *TET2* deficiency-related CH (TedCH).^4^ Our previous study suggest inflammation play a positive role in driving TedCH. To further explore the potential drivers of CH (i.e. LPS-induced inflammation), we assessed the impact of another four different environmental factors on TedCH and confirmed that accelerated TedCH depends on the establishment of an inflammatory environment (here colitis-induced).^5^

Competitive bone marrow transplantation assays were developed for analyzing TedCH in the chimeric mice and four different treatments were chosen for testing environmental impacts on TedCH (Fig. S1A). As shown in Figure S1B, C, expedited TedCH was observed upon dextran sulfate sodium salt (DSS) feeding compared with vehicle (Veh) feeding. Treatments with streptozotocin or additional irradiation appear to not affect TedCH while 5-fluorouracil (5-FU) has significant inhibition on TedCH (Fig. S1D–F, 2A–C).

Since DSS expedited TedCH in chimeric mice, we studied the underlying biological and pathological processes using *Tet2*-deficient primary mice, as they are more stable and can be monitored in large cohorts without irradiation. We turned to ask about the impact of chronic colitis by three cycles of low-dose DSS feeding (Fig. 1A). Consistently, even low-dose DSS feeding induced exacerbated colitis in *Tet2*-deficient primary mice (Fig. S3A–E). Upon DSS challenge, an increased disease score was observed and confirmed by the expression of gut barrier markers *Muc2* (mucin2) and *Ocln* (occludin) (Fig. S3F, H). Interestingly, *Tet2*-deficient primary mice appear to develop colitis spontaneously as shown by fluorescein 5-isothiocyanate staining in the serum and hematoxylin-eosin staining in the colon (Fig. S3G).

**Figure 1:**
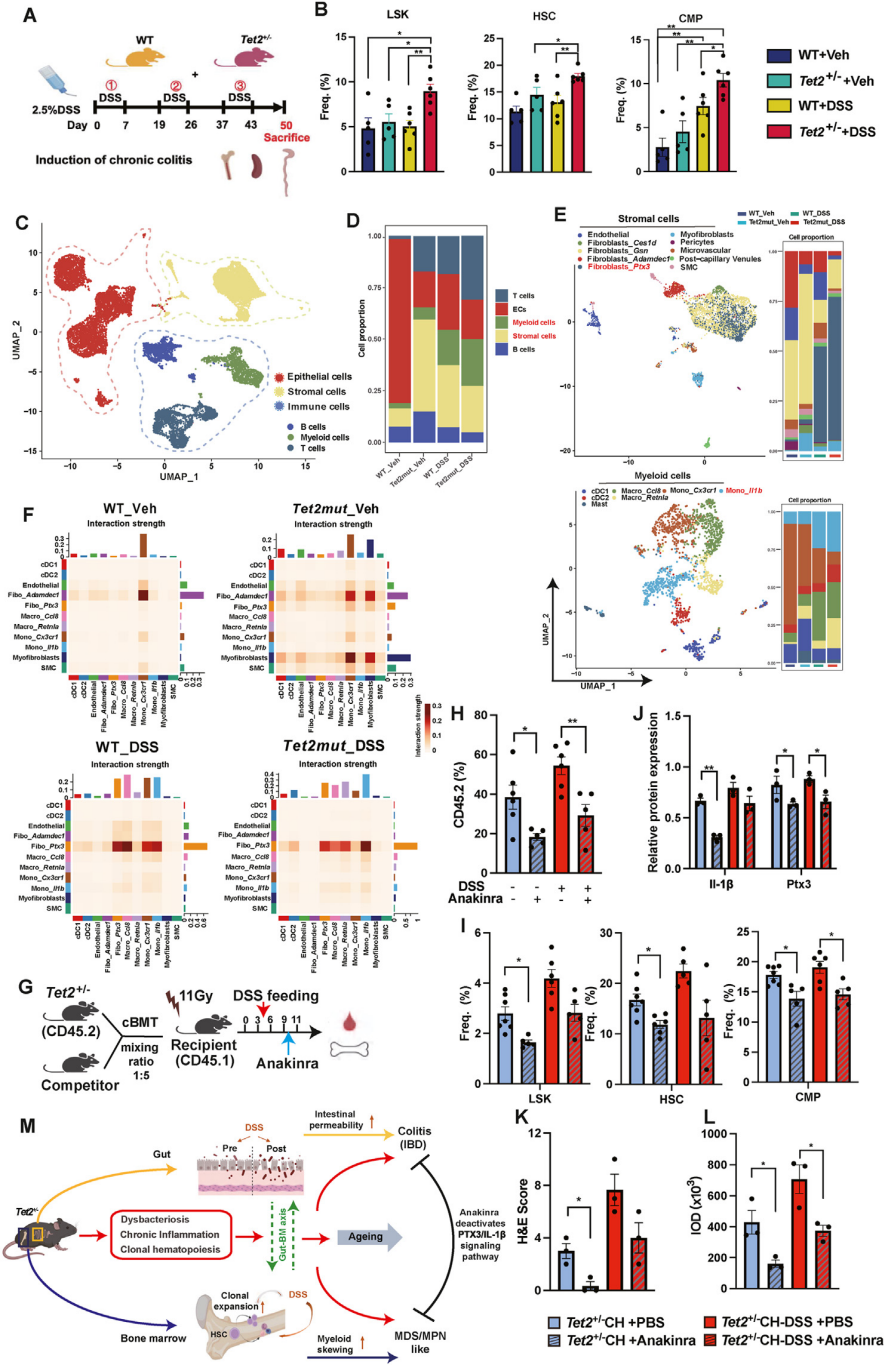
Clonal hematopoiesis cooperates with inflammation and dysbacteriosis in the gut-bone marrow axis to promote colitis and leukemia. **(A)** Experimental scheme for induction of chronic colitis by dextran sulfate sodium salt (DSS). Three cycles of DSS feeding were performed during the 50-day experimental procedure. Four animal groups were included in the entire procedure: WT_Veh, *Tet 2*^*+/−*^_Veh, WT_DSS, and *Tet2*^*+/−*^_DSS. **(B)** Quantifications of LSK (Lin^−^ Sca 1^+^ Kit^+^), HSC (hematopoietic stem cell), and CMP (common myeloid progenitor) in bone marrow (BM). **(C)** The UMAP plot showing five main populations in colon tissues. After lysis, all cells from colon tissues were subjected to single-cell RNA sequencing. A total of 18,092 high-quality cells with an average of about 3000 genes per cell were included in the UMAP plot. After quality control during the dataset analysis, the group of *WT_Veh* had 5589 cells, *Tet2mut_Veh* had 5649 cells, *WT_DSS* had 3382 cells, and *Tet2mut_DSS* had 4372 cells. The five main populations include epithelial cells (red) and stromal cells (yellow) and three major types of immune cells include myeloid cells (green), B cells (blue), and T cells (dark blue). **(D)** The stacking bar plot showing the portion of the five main annotated populations in each colon sample. **(E)** Stromal cells and myeloid cells were subjected to further clustering by UMAP as they had most significantly up-regulated biological pathways as indicated. The top panel is the UMAP plot of stromal cells and the stacking plot showing the potions of the subpopulations; the bottom panel is the UMAP plot of myeloid cells and the stacking plot showing the potions of the subpopulations. As indicated, ten subpopulations and seven subpopulations were annotated in stromal cells and myeloid cells respectively. **(F)** Heatmap of cell-to-cell talk strength between subpopulations of stromal cells and myeloid cells. **(G)** Schematic representation of anakinra treatment on the DSS-induced expedited TedCH. **(H)** Quantification of peripheral blood (PB) chimerism in TedCH mice treated with DSS or anakinra. **(I)** Quantification of hematopoietic stem/progenitor cells including LSK, HSC, and CMP in four groups of TedCH mice by flow cytometry. **(J)** The protein levels of IL-1β and Ptx3 were measured in colon tissues by Western blot. **(K)** Representative hematoxylin-eosin staining of colon tissue from each group and disease scores (damages in colon tissue) were quantified accordingly. **(L)** Semi-quantitative analysis of immunohistochemistry staining of Ptx3 based on integrated optical density (IOD). Scale bar, 100 μm. **(M)** We propose that the gut-bone marrow axis plays an important role in the co-symptoms of colitis and leukemia in the *Tet2*-mutant chimeric and primary mice. Dysbacteriosis, chronic inflammation, and clonal hematopoiesis are all assembled to promote the progression the co-symptoms over age. The PTX3/IL-1β signaling pathway plays an important role in promoting colitis and leukemia. The main text and supplemental information are provided for further discussion.

To clarify the DSS effect on hematopoiesis, cells from blood, spleen, and bone marrow were subjected to hematological cell counts, histology, and flow cytometry analysis. We observed skewed myelopoiesis trends in the *Tet 2*-deficient primary mice compared with wild-type controls (Fig. 1B; Fig. S4A–G).

Colon tissue from the four groups of mice was also used for single-cell RNA sequencing (scRNA-seq) analysis since this approach can provide a full map of cell-to-cell talk at the single-cell resolution. A total of five main populations including epithelial cells, stromal cells, and immune cells are annotated in the Uniform Manifold Approximation and Projection (UMAP) plot (Fig. 1C). However, *Tet 2*^*+/−*^_Veh, WT_DSS, and *Tet2*^*+/−*^_DSS appear to have a higher percentage of stromal cells compared with the WT_Veh control (Fig. 1D). Expression density of the respective markers for the five main cell populations is shown in Figure S5A. The overall enriched signaling pathway analysis suggests that the inflammatory signaling pathway is up-regulated in the myeloid cells of *Tet2*^*+/−*^_Veh, WT_DSS, and *Tet2*^*+/−*^_DSS compared with the WT_Veh control (Fig. S5B, 6A). Importantly, cell-cycle related pathway is also up-regulated in the myeloid cells of *Tet2*^*+/−*^_Veh compared with WT_Veh control (Fig. S5B). Interestingly, we also observed up-regulated epithelia-mesenchymal-transition pathway in the stromal cells of *Tet2*^*+/−*^_Veh, WT_DSS, and *Tet2*^*+/−*^_DSS compared with the WT_Veh control (Fig. S5B, 6A).

Stromal cells and myeloid cells from the colon UMAP pool were subject to further clustering to distinguish sub-populations. In total, eight sub-populations of cells in the stromal cells and seven sub-populations of cells in the myeloid cells were annotated according to the typical marker gene expression (Fig. 1E; Fig. S6B). Expression of *Ptx3* (pentraxin 3) and *Il-1β* (interleukin 1 beta) is plotted in the UMAP space of colon tissue, stromal cells, or myeloid cells as indicated (Fig. S5C). To our surprise, expression of *Ptx3* appears to be specific to stromal cells in the colon while expression of *Il-1β* appears to be specific to myeloid cells in the colon in the scRNA-seq dataset. Five sub-populations from the stromal cells and six sub-populations from the myeloid cells had the most alterations in the percentage and therefore were used for calculating cell-to-cell talk strength. Upon DSS feeding, the interaction between *Ptx3*^*+*^ stromal cells and several myeloid cells was dramatically activated (Fig. 1F).

As shown in Figure S5D–G, IL-1β/IL1-R1 signaling is actively involved in the cell–cell interaction, associated with the expression of many downstream genes encoding complement protein proteins or inflammatory ligands (*C1ra*, *C1s1*, *C3*, and *Cxcl12*). Surprisingly, *Il1r1* along with *C1ra*, *C1s1*, *C3*, and *Cxcl12* all were dominantly expressed in the stromal cells of the colon compared with the other four main cell populations (Fig. S5H).

To guarantee that PTX3/IL-1β signaling is involved in human colitis or leukemia, we searched publicly available datasets of bulk RNA-seq or scRNA-seq and compared their expression between the disease group and healthy controls. Data shown in Figure S7A–D suggest that increased expression of *PTX3* is detected in the myeloid leukemia group (datasets with patients with acute myeloid leukemia or myelodysplastic syndromes were analyzed). Interestingly, mutations in *TET2* appear to have higher expression of *PTX3* as well (Fig. S7A, D). Importantly, higher expression of *PTX3* had a worse prognosis in the TCGA-LAML cohort while expression of *IL-1β* failed to stratify the cohort (Fig. S7B, C). Additionally, analysis of colitis samples suggests that higher expression of *PTX3* and *IL-1β* took place in some cohorts of colitis samples compared with the controls (Fig. S7E–G).

As we observed that *Ptx3* and *Il-1r1* were dominantly (if not specifically) expressed stromal cells of colon tissue in mice (Fig. S10A, B), we characterized if this pattern was conserved in humans. We downloaded the publicly available scRNA-seq datasets of colitis and re-analyzed the cells.^5^ Results from Figure S7H–M and Figure S8 suggest that the percentage of *PTX3*^*+*^ fibroblasts indeed increased according to the grade of colitis. Although partial myeloid cells express *PTX3* themselves, *PTX3* is also dominantly expressed in stromal cells.

We postulate that the PTX3/IL-1β signaling pathway is present at the protein level in the colon of *Tet 2*^*+/−*^ mice with chronic inflammation. To validate this hypothesis, we performed Western blot analysis to examine the expression of Il-1β and Ptx3 (Fig. S10C, D). Immunohistochemical staining revealed high levels of Ptx3 expression in inflammatory cells infiltrating the colonic tissues of *Tet2*^*+/−*^ mice (Fig. S10E, F).

As inhibitors specific to PTX3/Ptx3 activity are not available, we turn to use IL1R1-specific inhibitor anakinra to assess the function of Ptx3 in the TedCH, colitis, and aberrant hematopoiesis. Competitive bone marrow transplantation assays for TedCH were conducted along with DSS and/or anakinra treatment (Fig. 1G). Consistent with its anti-inflammation function, the application of anakinra mitigated TedCH expansion in the mice fed with vehicle or DSS (Fig. 1H; Fig. S10G). Aberrant hematopoiesis also appears to be partially rescued in the TedCH_Anakinra mice compared with the TedCH_PBS mice (Fig. 1I; Fig. S9A, 10H, K). Importantly, the application of anakinra reduced the expression of both Ptx3 and Il-1β (Fig. 1J; Fig. S9B). The reduced expression of Ptx3 upon anakinra treatment is also validated by immunohistochemistry; the pathologic index is also largely ameliorated upon anakinra treatment (Fig. 1K, L; Fig. S9C, D).

In summary, the present study demonstrates that the gut-bone marrow axis plays an important role in the co-symptoms of colitis and leukemia in the *Tet2*-mutant chimeric and primary mice. Dysbacteriosis, chronic inflammation, and clonal hematopoiesis are all assembled to promote the progression of the co-symptoms over time. Our single-cell transcriptomic analysis highlights the gut *Ptx3*^*+*^ fibroblasts in driving the entire disease. Mitigation of inflammation by the IL-1R1 inhibitor anakinra can down-regulate Ptx3, repair the gut barrier, and slow down TedCH. The study demonstrates that PTX3/IL-1β signaling-associated clonal hematopoiesis plays important roles in the gut-bone marrow axis and related colitis and leukemia (Fig. 1M).

## Ethics declaration

The animal experimentation was performed in accordance with protocols approved by the Animal Care and Use Committee of Tianjin Medical University.

## CRediT authorship contribution statement

**Hang He:** Validation, Visualization, Writing – original draft, Writing – review & editing. **Yuchen Wen:** Validation, Visualization, Writing – original draft, Writing – review & editing. **Qingran Huo:** Visualization. **Hanzhi Yu:** Validation, Visualization. **Jingjing Liu:** Validation. **Wenyan Jin:** Validation, Visualization. **Zhiqin Wang:** Validation, Visualization. **Huaquan Wang:** Methodology, Writing – review & editing. **Zhigang Zhao:** Project administration, Writing – review & editing. **Zhigang Cai:** Funding acquisition, Methodology, Writing – original draft, Writing – review & editing.

## Conflict of interests

Z.C. is a scientific advisor to Beijing SeekGene BioSciences Co. Ltd., Beijing, China. The other authors declared no potential conflict of interests.

## Funding

This work was supported in part by grants from the Tianjin Medical University Talent Program of China and from the National Science Foundation of China (No. 82170173, 82371789 to Z.C.).

## Data availability

The raw sequencing data from this study have been deposited in the Genome Sequence Archive in BIG Data Center (https://bigd.big.ac.cn/), Beijing Institute of Genomics (BIG), Chinese Academy of Sciences, under the accession number PRJCA016651 (the datasets will be available to the public once the manuscript in press).
