# Supplementary material for: Cooperative progression of colitis and leukemia modulated by clonal hematopoiesis via PTX3/IL-1β pro-inflammatory signaling
Source: Genes Dis. 2024 Aug 23;12(4):101397. doi: 10.1016/j.gendis.2024.101397 (PMC11995054; doi:10.1016/j.gendis.2024.101397)
Supplement: Multimedia component 1 [file mmc1.docx]

**Supplemental Material**

**Materials and Methods**

***Mice***

We used wild-type C57BL/6 (CD45.2), C57BL/6.SJL (CD45.1, BoyJ) and *Tet2^+/-^* mice at the age of 8 weeks ^1^. Animal experimentation was performed in accordance with protocols approved by the Animal Care and Use Committee of Tianjin Medical University.

***Competitive bone marrow transplantation and In vivo assays***

Recipient animals (F1, CD45.2/ CD45.1) were lethally irradiated (7Gy plus 4Gy) one day prior to transplantation (intravenous tail injection) of donor cells. For generating chimeric mice mimicking hematopoietic clonal expansion, *Tet2^+/-^* donor cells and BoyJ donor cells were mixed at a ratio of 1: 5 (100K: 500K). 4 weeks after BM transplantation animals were injected with 5-FU (5-fluorouracil F6627, Sigma-Aldrich) at a dose of 150 mg/kg in 100ul of PBS. Mice were given a single intraperitoneal injection with a dose of 180 mg/kg STZ (streptozocin, BS1485, Biosharp) after fasting for more than 12 hours to establish hyperglycemia model. Mice were treated with ddW with or without 2.5% DSS (dextran sulfate sodium, MP Biomedicals) for 1 week, ddW for another week for three cycles. For pharmacological assays, Anakinra (GC39339, GLPBIO) was injected intra-peritoneally at a dose of 37ug/mouse in 100ul of PBS every other day for 1 month.

***Flow cytometry***

The BM cells were flushed out with FACS buffer. Single-cell suspensions were treated with red blood cell lysis buffer, stained, and analyzed using FACS Canto II (BD Biosciences). For mature cell analysis, staining with antibodies against B220 (#103208, BioLegend), CD3 (#100206, BioLegend), Gr-1 (#108408, BioLegend), CD11b (#101208, BioLegend). For early hematopoietic cell analysis, cells were incubated with biotinylated antibodies against the lineage (Lin) markers: CD11b, Ter119 (#116208, BioLegend), Gr-1, CD3, B220, and the fluorescence-conjugated antibodies: c-Kit (#105812, BioLegend), Sca-1 (#108126, BioLegend), CD34 (#11-0341-85, BioLegend), CD150 (#115912, BioLegend), CD48 (#103424, BioLegend), and CD16/32 (#101318, BioLegend).

***Single cell RNA-sequencing***

Immature hematopoietic cells (Linage-negative, Lin^-^) was collected by EasySep^TM^ cell separation kit (Stem Cell Co.). After harvested, cell count and viability of the bone marrow were estimated using fluorescence Cell Analyzer (Countstar^®^ Rigel S2) with AO/PI reagent after removal erythrocytes (R1010, Solarbio) and then dead cells removal was decided to be performed or not (130-090-101, Miltenyi). Finally fresh cells were washed twice in the RPMI1640 and then resuspended at 1×10^6^ cells per ml in 1×PBS and 0.04% bovine serum albumin.

Colon tissues were washed in ice-cold RPMI1640 and dissociated using Collagenase Ⅰ (V900891-100MG, Sigma) and Collagenase Ⅳ (C5138-500MG, Sigma). DNase Ⅰ (9003-98-9, Sigma) treatment was optional according to the viscosity of the homogenate. Cell count and viability was estimated using fluorescence Cell Analyzer (Countstar^®^ Rigel S2) with AO/PI reagent after removal erythrocytes (R1010, Solarbio) and then dead cells removal was decided to be performed or not (130-090-101, Miltenyi). Finally fresh cells were washed twice in the RPMI1640 and then resuspended at 1×10^6^ cells per ml in 1×PBS and 0.04% bovine serum albumin.

Single-cell RNA-Seq libraries were prepared using SeekOne^®^ MM Single Cell 3’ library preparation kit (No.K00104, SeekGene). Briefly, the appropriate number of cells were loaded into the flow channel of SeekOne^®^ MM chip which had 170,000 microwells and allowed to settle in microwells by gravity. After removing the unsettled cells, sufficient Cell Barcoded Magnetic Beads (CBBs) were pipetted into flow channel and also allowed to settle in microwells with the help of a magnetic field. Next excess CBBs were rinsed out and cells in MM chip were lysed to release RNA which was captured by the CBB in the same microwell. Then all CBBs were collected and reverse transcription were performed at 37℃ for 30 minutes to label cDNA with cell barcode on the beads. Further Exonuclease I treatment were performed to remove unused primer on CBBs. Subsequently, barcoded cDNA on the CBBs was hybridized with random primer which had reads 2 SeqPrimer sequence on the 5’ end and could extend to form the second strand DNA with cell barcode on the 3’ end. The resulting second strand DNA were denatured off the CBBs, purified and amplified in PCR reaction. The amplified cDNA product was then cleaned to remove unwanted fragments and added to full length sequencing adapter and sample index by indexed PCR. The indexed sequencing libraries were cleanup with SPRI beads, quantified by quantitative PCR (KK4824, KAPA Biosystems) and then sequenced on illumina NovaSeq 6000 with PE150 read length.

***Fecal microbiota evaluation***

Mice were individually placed in clean cages for feces collection. Fresh fecal samples were collected into sterile cryopreservation tubes, quickly frozen in liquid nitrogen and stored at −80°C. Purified amplicons were pooled in equimolar and paired-end sequenced on an Illumina MiSeq PE300 platform/NovaSeq PE250 platform (Illumina, San Diego,USA) according to the standard protocols by Majorbio Bio-Pharm Technology Co. Ltd. (Shanghai, China).

***H&E Staining and Immunohistochemistry Staining***

The tissue samples were ﬁxed in 4% paraformaldehyde. After the dehydration in ethanol, the tissue was embedded in paraﬃn then sectioned with a thickness of 4 μm. The histopathological feature was tested via H&E staining. To perform immunohistochemistry staining, the tissue sections were deparaﬃnized, rehydrated, and rinsed, followed by antigen retrieval and blocking (goat serum). Next, the tissue sections were incubated with primary antibody overnight and followed by the biotinylated secondary antibodies. The DAB Horseradish Peroxidase Color Development Kit (Dako, Agilent Technologies, USA) was applied for color reaction in immunohistochemistry staining. Finally, the tissue sections were observed under the optical microscope or ﬂuorescence microscope. The Image J software was applied for scoring the immunohistochemistry picture.

Immunohistochemical (IHC) staining was using a Leica Bond RX stainer (Leica, Buffalo Grove, IL). Slides were retrieved for 20 min using Epitope Retrieval 1 (Citrate; Leica) and incubated in Protein Block (Dako, Agilent, Santa Clara, CA) for 5 min. Primary antibodies were diluted in Background Reducing Diluent (Dako) as follows: Ptx3 (41372, Sabbiotech, USA) at 1:900, which was diluted in Bond Diluent (Leica) at 1:200. Primary antibodie was diluted in Background Reducing Diluent (Dako) and incubated for 15 min. Immunostaining visualization was achieved by incubating slides 10 min in DAB and DAB buffer from the Bond Polymer Refine Detection System. Slides were counterstained for 5 min using Schmidt hematoxylin, followed by several rinses in 1x Bond wash buffer and distilled water.

***Western blot analysis***

Proteins were harvested from cells and colon tissues with RIPA Lysis Buffer (Beyotime, Jiangsu, China) supplemented with phenylmethyl sulfonyl fluoride (PMSF) protease inhibitor and phosphatase inhibitor. Total protein concentration was determined by BCA Protein Assay Kit (Beyotime, Jiangsu, China), denatured protein samples of appropriate quality of proteins were subjected to sodium dodecyl sulfate polyacrylamide gel electrophoresis (SDS-PAGE) and then transferred to PVDF membranes. Then membranes were later blocked with 5% skimmed milk, and incubated were immunodetected with specific antibodies against Il-1β (515598; SANTA, USA), Ptx3 (41372, Sabbiotech, USA), and GAPDH (ab181620, Abcam, USA) overnight at 4 °C. Protein bands were visualized by the MINICHEMI Imaging System (Surwit, Hangzhou, China) using the commercial Pierce ™ Fast Western Blot Kit and the ECL Substrate (GenStar, Beijing, China).

**List of computation & visualizing software for mouse scRNA-seq**

Most of the software used for computing and visualizing the scRNA-seq of both mouse and human have been described in our previous study^2^. Coding scripts is available upon request.

***Statistical analysis***

Statistical analysis was conducted by using GraphPad 9. Comparisons between 2 groups were determined by using a two-tail student’s t-test. Comparison of multiple groups were determined by using an ANOVA analysis of variance with the Dunnett multiple comparisons test. Most of experiments in this study were repeated 2 or 3 times independently and representative data was shown. Results with p < 0.05 were considered statistically significant.

**Reference**

1. Li Z, Cai X, Cai C-L, et al. Deletion of Tet2 in mice leads to dysregulated hematopoietic stem cells and subsequent development of myeloid malignancies. *Blood, The Journal of the American Society of Hematology*. 2011;118(17):4509-4518.

2. He H, Wang Z, Yu H, Zhang G, Wen Y, Cai Z. Prioritizing risk genes as novel stratification biomarkers for acute Monocytic leukemia by integrative analysis. *Discover Oncology*. 2022;13(1):55.

**Figures**

**
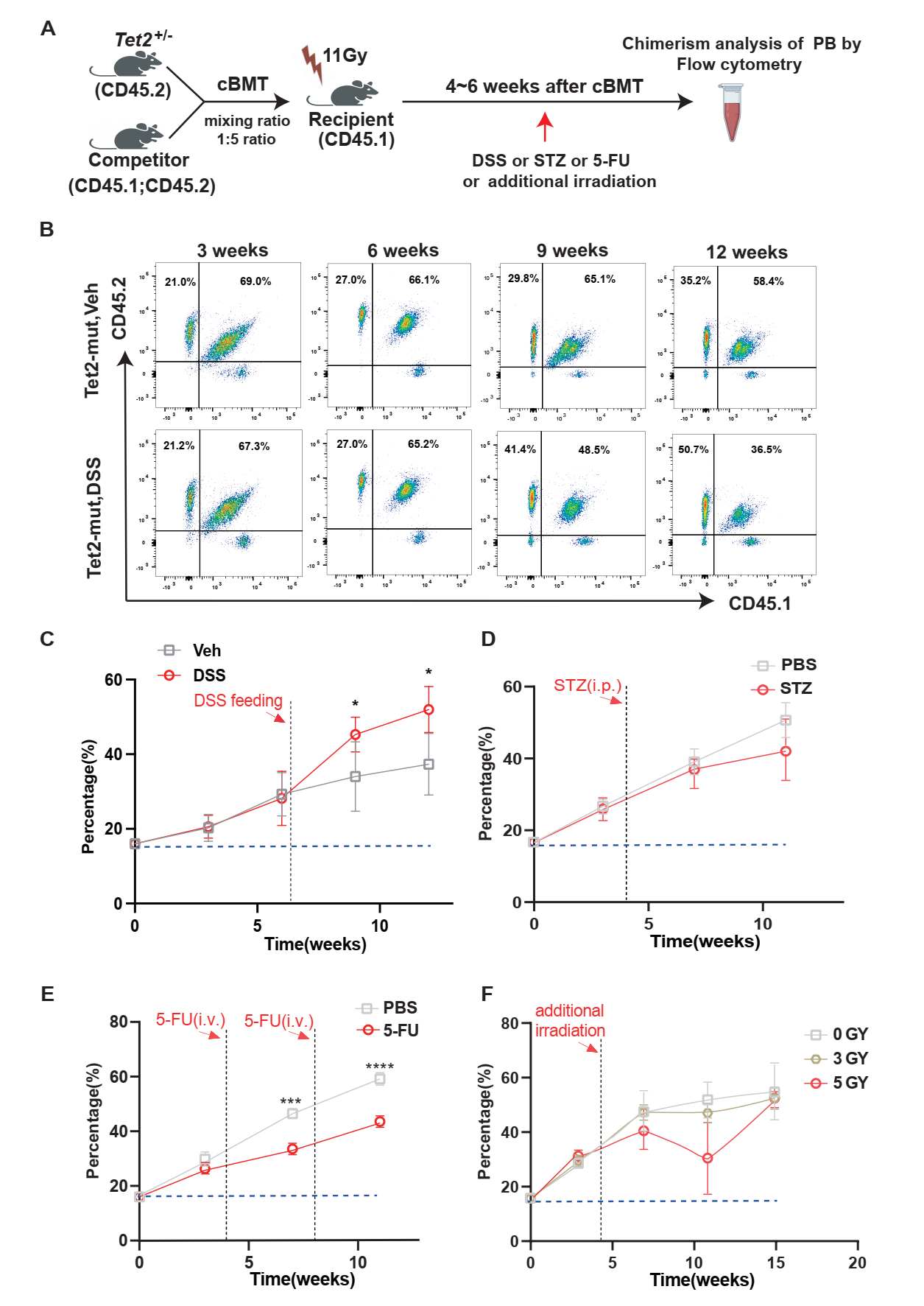
**

**Figure S1 Assessing impacts of various treatments on TedCH trajectory.**

(A-C) Representative flow profiles of PB from the chimeric mice treated with STZ (A), or 5-FU (B), or additional irradiation (C) as indicated.

(D-) Quantified trajectories of TedCH in the chimeric mice treated with STZ (C), or 5-FU (D), or additional irradiation (E) as indicated.

Data are shown as means ± SEMs. Number of biological repeats (animals): n = 5~7. Experiments of DSS treatment were repeated twice. Experiments of STZ or 5-FU or additional irradiation treatment were performed once. *, p < 0.05; **, p < 0.01; ***, p < 0.001; ****, p < 0.0001.

**
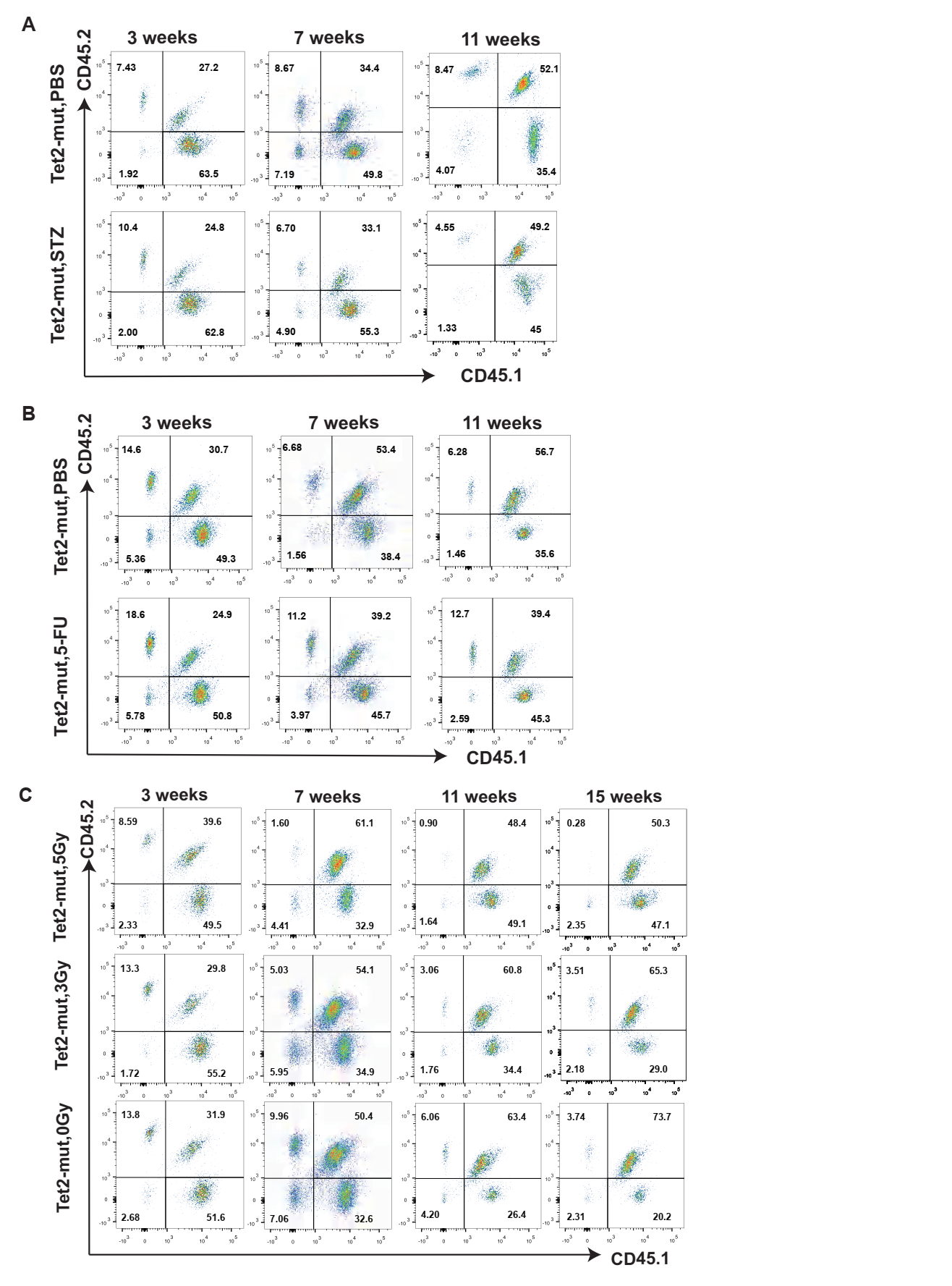
**

**Figure S2 Assessing impacts of various treatments on TedCH trajectory**

(A) Competitive bone marrow transplantation (cBMT) assays were used to search for cooperative environmental factors of TedCH. See Methods for detailed cBMT experimental procedures. Treatments were applied once TedCH is set up at the 3~6 weeks post cBMT. Results of four different treatments (DSS, STZ, 5-FU and additional irradiation) were included in this study. As shown in the Figure 1A, DSS accelerated TedCH. Expedited TedCH was also observed when *Tet2^+/-^* HSPCs were mixed with genetically stable inflammatory HSPCs and the detail results will be reported in another study (Wen and Cai *et al*., manuscript in preparation, 2024). cBMT, competitive bone marrow transplantation; PB, peripheral blood.

(B and C) Representative flow profiles of PB from the chimeric mice fed with DSS or normal water (vehicle, veh) (B) and the quantified trajectories of TedCH in the chimeric mice (C).

(D-F) Quantified trajectories of TedCH in the chimeric mice treated with STZ (D), or 5-FU (E), or additional irradiation (F) as indicated.

Data are shown as means ± SEMs. Number of biological repeats (animals): n = 5~7. Experiments of DSS treatment were repeated twice. Experiments of STZ or 5-FU or additional irradiation treatment were performed once. *, p < 0.05; **, p < 0.01; ***, p < 0.001; ****, p < 0.0001.

**
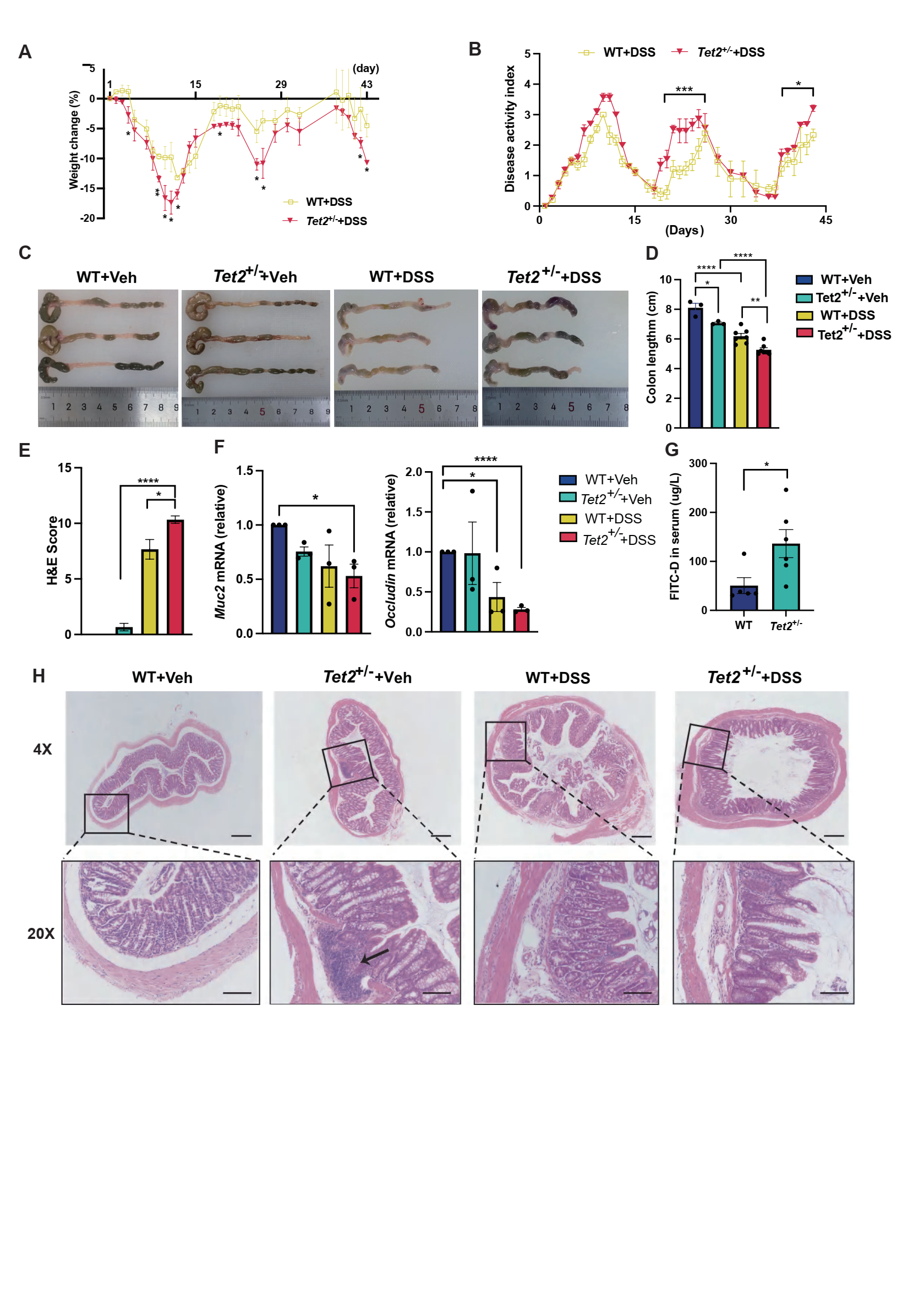
**

**Figure S3 *Tet2*-deficient mice manifest exacerbated colitis and aberrant microbiota composition when fed with DSS.**

(A and B) Body weights and disease scores (disease activity index, DAI) were monitored daily and plotted during the induction.

(C-E) *Tet2*-deficient mice manifest exacerbated colitis based on colon pathology. Colons from the 4 groups of mice were photographed (C) and quantified based on colon length (D). Damage scores of colons were monitored and plotted based on H&E staining (E).

(F) Expression of two classic gut barrier markers *Muc2* and *Occludin* in colon were quantified by qRT-PCR.

(G) Damage of gut barrier were quantified by FITC-dextran staining in serum of PB.

(H) Images of hematoxylin and eosin (H&E) staining of spleen in each group after DSS treatment. (Scale bar: 4x, 500μm; 20x, 100μm).

Data are shown as means ± SEMs in A to H. Number of biological repeats (animals): n = 3~7. Experiments of DSS treatment on primary mice were repeated three times. *, p < 0.05; **, p < 0.01; ***, p < 0.001; ****, p < 0.0001.

**
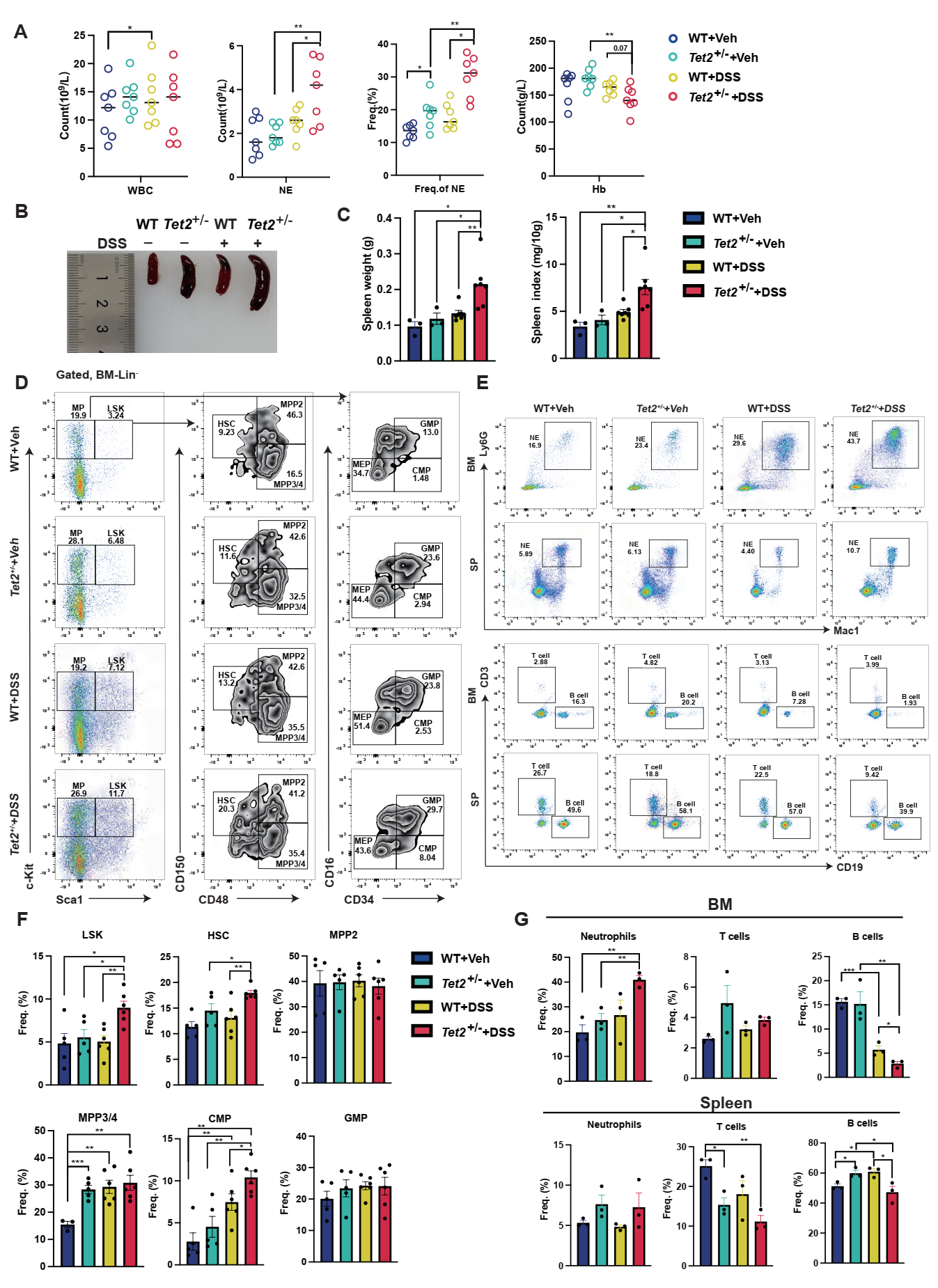
**

**Figure S4 Chronic DSS feeding induces exacerbated myelopoiesis in *Tet2*-deficient mice revealed by flow cytometry.**

(A) Hematological parameters of PB were monitored at the end-point of the DSS induction.

(B and C) Photography and quantification of spleens.

(D and F) Gating strategies, representative flow profiles and quantifications of HSPCs in BM. LSK, Lin^-^;Sca1^+^;Kit^+^. HSC, hematopoietic stem cell. MPP, multiple potent progenitor. CMP, common myeloid progenitor. GMP, granulocyte-monocyte progenitor.

(E and G) Quantification of mature cells including neutrophils, T cells, and B cells in bone marrow and spleen. BM, bone marrow; SP, spleen; PB, peripheral blood.

Data are shown as means ± SEMs. Number of biological repeats (animals): n = 3~7. Experiments of DSS treatment on primary mice were repeated three times. *, p < 0.05; **, p < 0.01; ***, p < 0.001; ****, p < 0.0001.

**
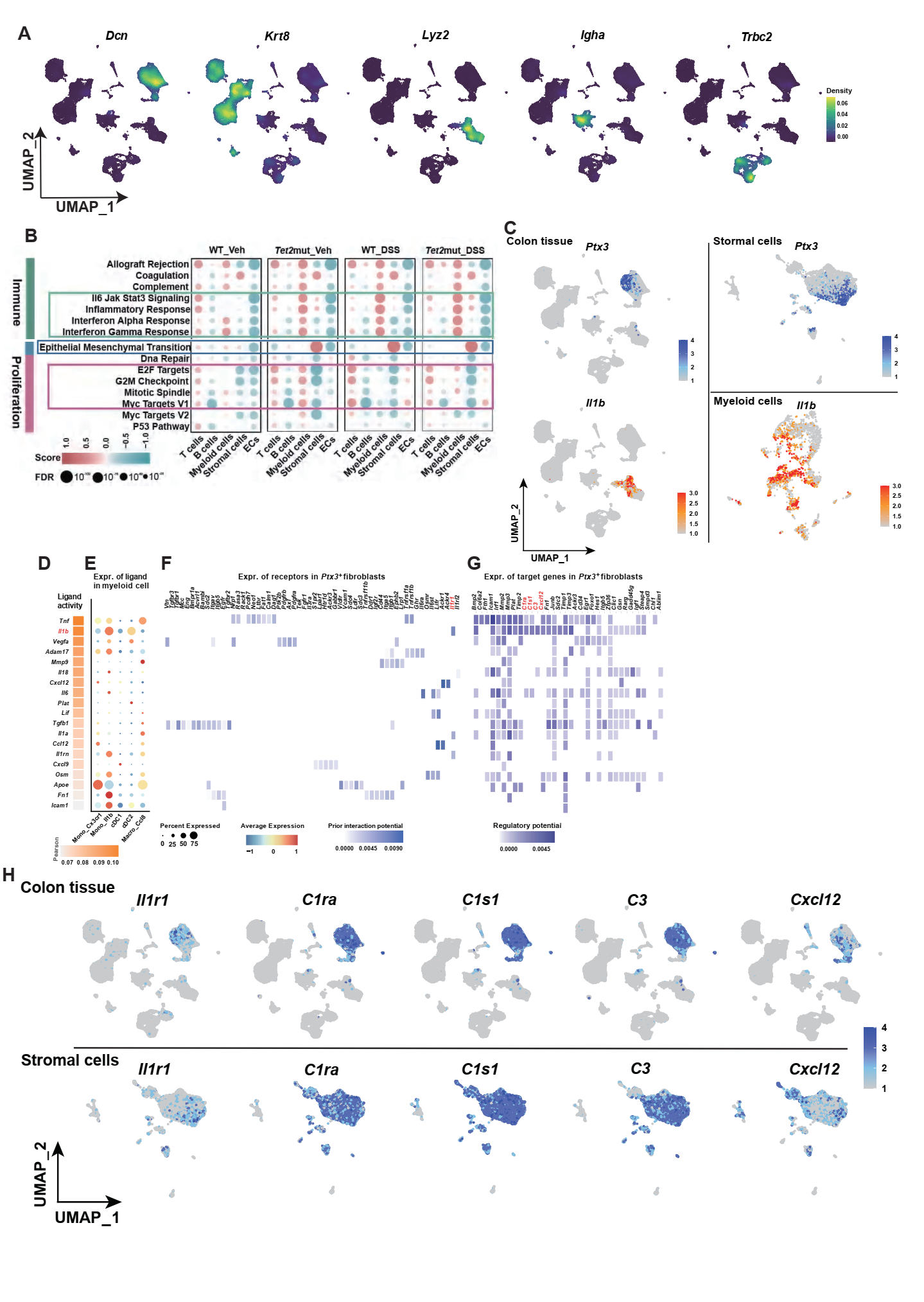
**

**Figure S5 Single-cell RNA-seq analysis of colon tissue from *Tet2*-deficient mice when feed with DSS or vehicle.**

(A) Expression of representative annotation markers for the 5 main cell populations in the UMAP plot of colons. *Dcn* for stromal cells; *Krt*8 for epithelial cells; *Lyz2* for myeloid cells; *Igha* for B cells; and *Trbc2* for T cells.

(B) Heatmap of dysregulated biological pathways in the 5 populations of colon tissues from 4 groups of samples.

(C) Expression of *Ptx3* and *Il-1β* in the colon tissues (left panel) or in stromal cells (up-right panel) or in myeloid cells (bottom-right panel), respectively.

(D-G) Detailed molecular events of the cell-to-cell talk between myeloid cells and stromal cells. Of note, *Ptx3^+^* ﬁbroblasts and *IL-1β ^+^* monocytes appear to be most involved. *IL-1β ^+^* monocytes express numerous ligands including *IL-1β* (D and E). *IL-1β* binds *Il1-1r1* in *Ptx3^+^* ﬁbroblasts and stimulates a couple of downstream genes (F and G).

(H) Expression of *IL-1β* receptor *Il1r1* and relevant genes encoding complement components or inflammation regulators (*C1ra*, *C1s1*, *C3* and *Cxcl12*) were plotted on UMAP of colon tissue (top panel) or on the UMAP plot of stromal cells (bottom panel).

**
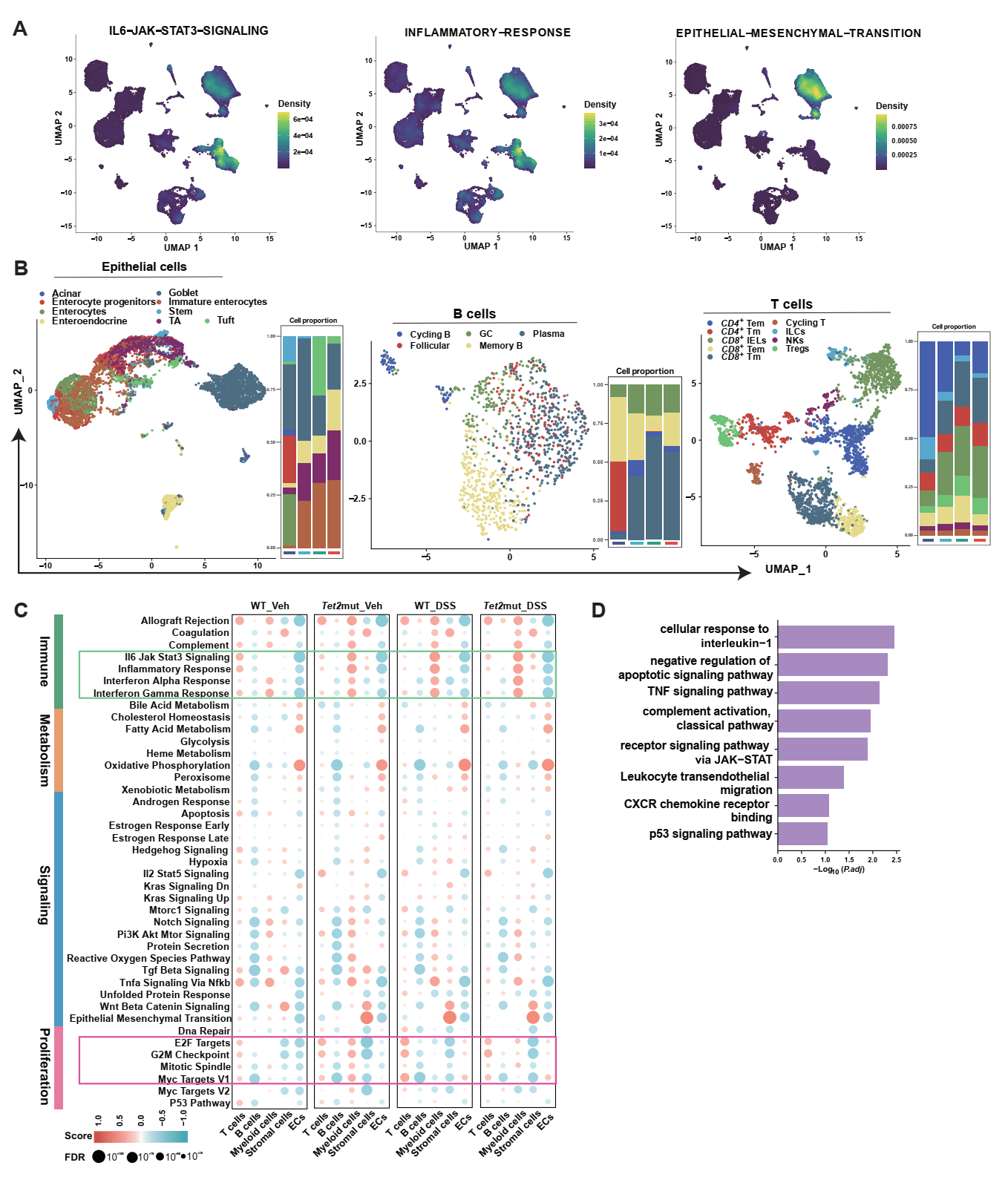
**

**Figure S6 Characterization of colon cells in *Tet2^+/-^* mice after chronic infection**

(A) UMAP visualization of enrichment scores for specific gene sets.

(B) UMAP showing the composition of epithelial cells, B cells and T cells colored by cluster (left), and Bar plot showing the changes in the cell proportion of each group (right).

(C) Heatmap of dysregulated biological pathways in the 5 populations of colon tissues from 4 groups of samples.

(D) Representative KEGG pathways enrichment of the predicted target genes expressed in *Ptx3^+^* ﬁbroblasts.

**
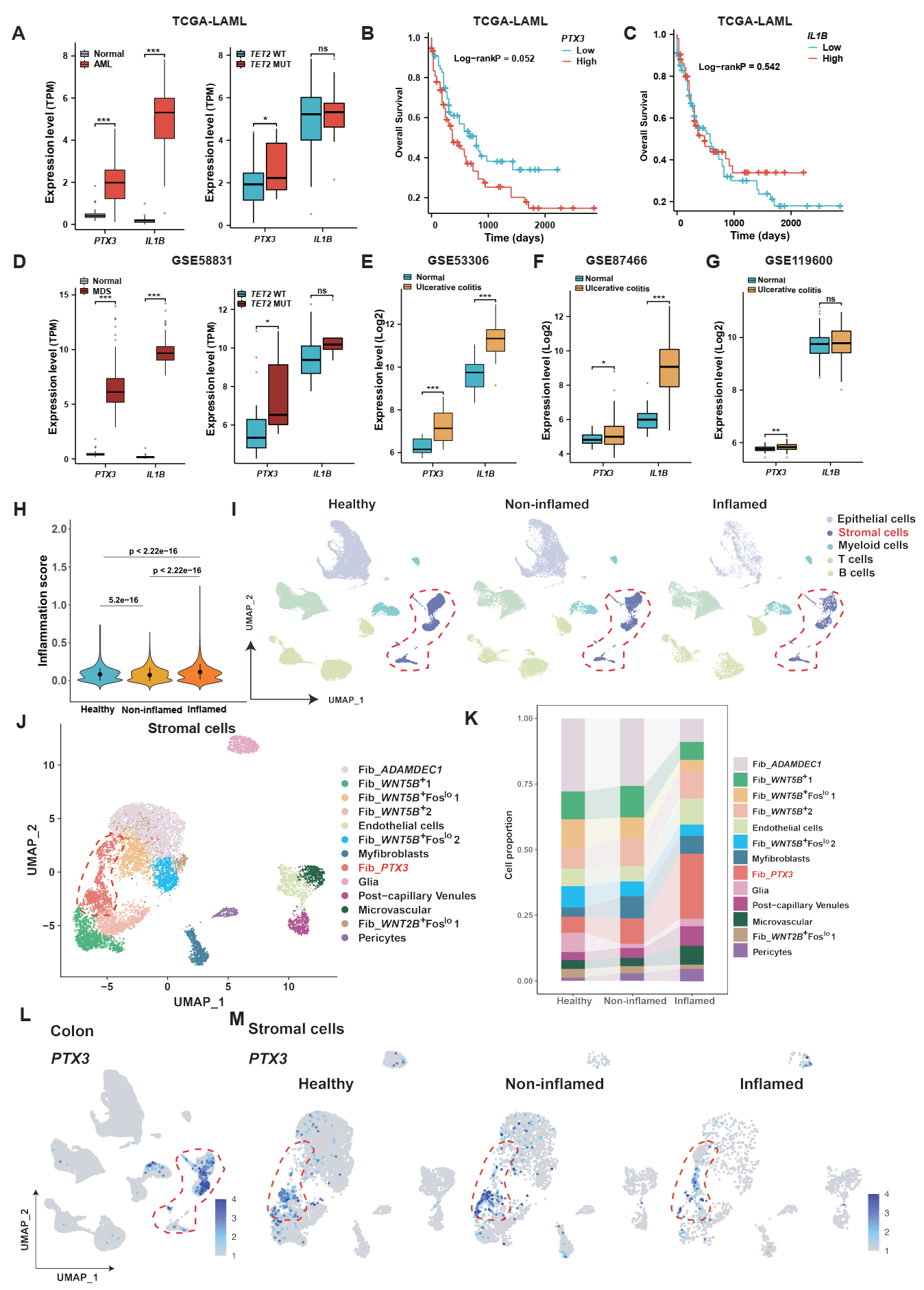
**

**Figure S7 Aberrant PTX3/IL-1β signaling is indicated in human leukemia or colitis through analysis of clinical samples.**

(A) Expression of *PTX3* and *IL-1β* in unsorted AML patients (left panel) or in AML patients carrying *TET2* mutation (right panel). Bulk RNA-seq datasets of bone marrow were included in the analysis.

(B-C) Prognosis value of marker genes *PTX3* and *IL-1β* in the TCGA-LAML cohort.

(D) Expression of *PTX3* and *IL-1β* in unsorted MDS patients (left panel, bone marrow samples) or in MDS patients carrying *TET2* mutation (right panel, bone marrow samples).

(E-G) Expression of *PTX3* and *IL-1β* in patients with ulcerative colitis and healthy controls, based on three independent cohorts. Bulk RNA-seq datasets of colon tissues were included in E and F; Bulk RNA-seq dataset of blood were included in G.

(H-M) Aberrant expression of *PTX3* and *IL-1β* was also detected using scRNA-seq dataset of human colitis samples. The dataset is downloaded from the website Broad Data Use Oversight System (https://duos.broadinstitute.org) and re-analyzed. The cohorts include two groups of human colon samples: colons from healthy controls (n=10) and colons from ulcerative colitis patients (n=7). Patient samples from both non-inflamed regions and inflamed regions were n=7 respectively. Inflammation scores suggest the three groups has significant difference overall (H). UMAP plots of the human colon scRNA-seq dataset also show five main populations as similar as Figure 1C: epithelial cells, stromal cells and three immune cells (myeloid cells, B cells and T cells) (I). Stromal cells were highlighted by the dotted lines (I). Stromal cells were further annotated by UMAP plots and *PTX3^+^* fibroblasts were screened out by dotted lines (J). Of note, stacking plots indicate percentage of *PTX3^+^* fibroblasts were increased according to the inflammation grade of the colitis (K). Expression of *PTX3* is shown in the UMAP plot of colon tissue (L) and three groups of stromal cells (M) as indicated.

**
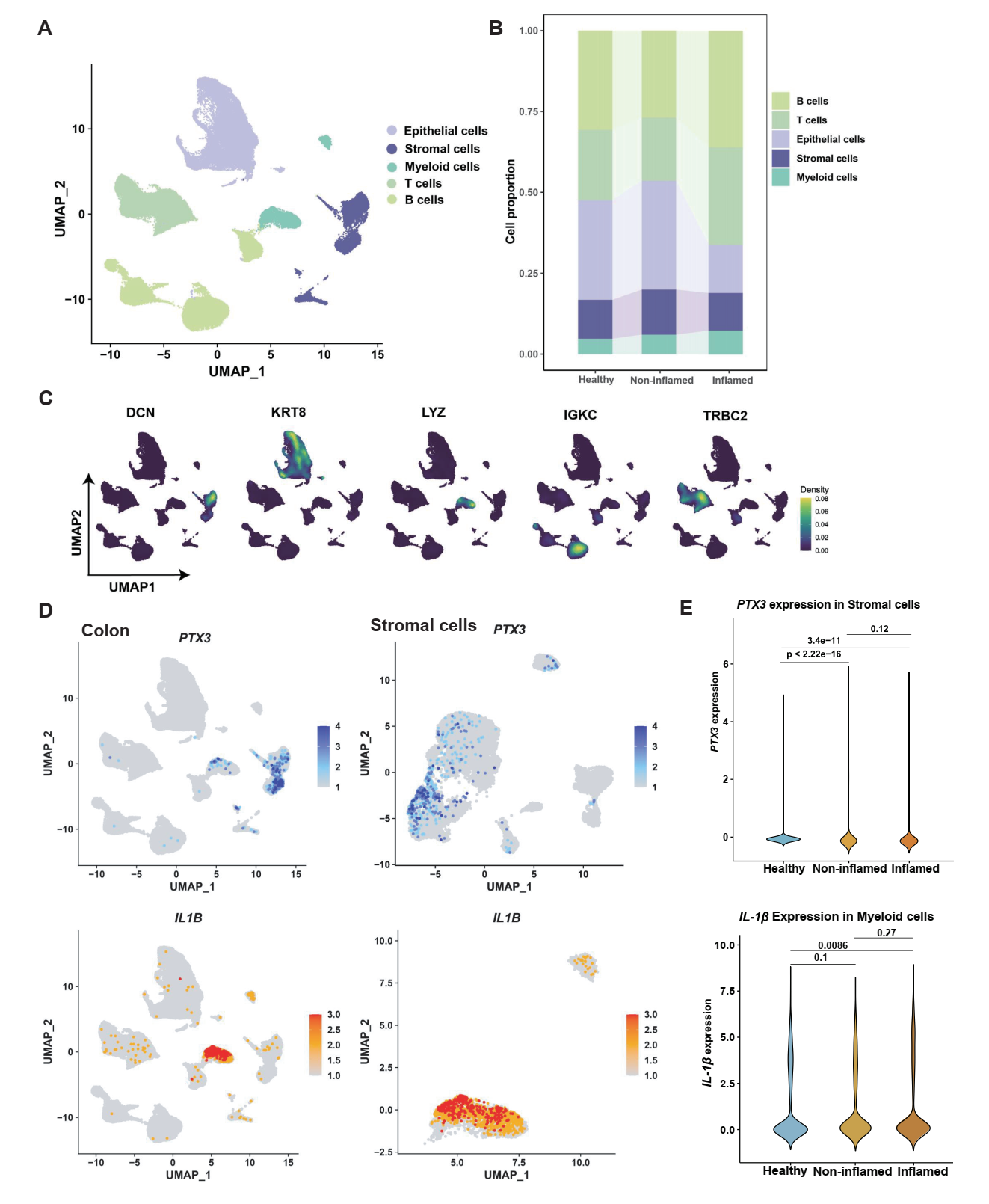
**

**Figure S8 Aberrant PTX3/IL-1β signaling is indicated in human colitis through analysis of clinical samples, related to Figure 5.**

(A) UMAP plot showing 5 main populations in colon tissues. A total of 90454 high-quality cells with an average of about 3000 genes per cell were included in the UMAP plot. After quality control during the dataset analysis, the group of *Health* has 55897 cells; *Non-inflamed* has 20188 cells; *Inflamed* has 14369 cells.

(B) Stacking bar plot showing the portion of the 5 main annotated populations in each colon sample.

(C) Expression of representative annotation markers for the 5 main cell populations in the UMAP plot of colons. *DCN* for stromal cells; *KRT*8 for epithelial cells; *LYZ* for myeloid cells; *IGKC* for B cells; and *TRBC2* for T cells.

(D) Expression of *PTX3* and *IL-1β* in the colon tissues (left panel) or in stromal cells (up-right panel) or in myeloid cells (bottom-left panel), respectively.

(E) Violin plots showing the expression of *PTX3* in the stromal cells (up panel) or in stromal cells (bottom panel).

**
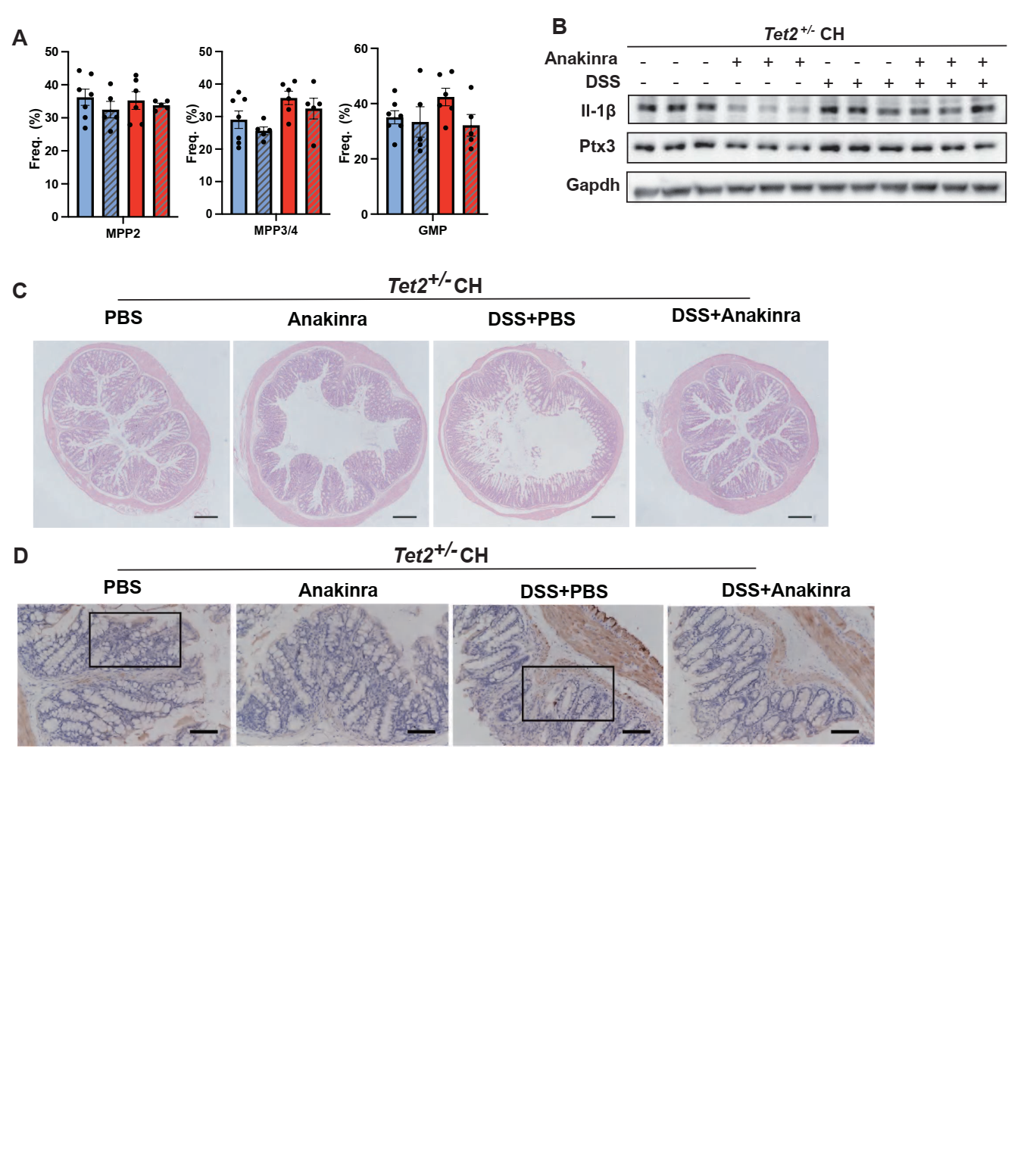
**

**Figure S9 Pharmacological blockage of IL-1β signaling by Anakinra inhibits Ptx3 expression and inflammation in colon and mitigates TedCH in blood.**

(A) Quantification of HSPCs including MPP2, MPP3/4 and GMP in 4 groups of TedCH mice by flow cytometry.

(B) The protein levels of IL-1β and Ptx3 were measured in colon tissues by western blot.

(C) Representative H&E staining of colon tissue from each group and disease scores (damages in colon tissue) were shown accordingly. Scale bar is 500 μm.

(D) Representative immunohistochemical (IHC) staining of Ptx3 in colon of immunohistochemistry staining of Ptx3. scale bar is 100 μm.

Data are shown as means ± SEMs in A-D. Number of biological repeats (animal mouse): n = 5~7. Experiments of DSS treatment combined with Anakina on TedCH mice were repeated twice. *, p < 0.05; **, p < 0.01; ***, p < 0.001; ****, p < 0.0001.

**
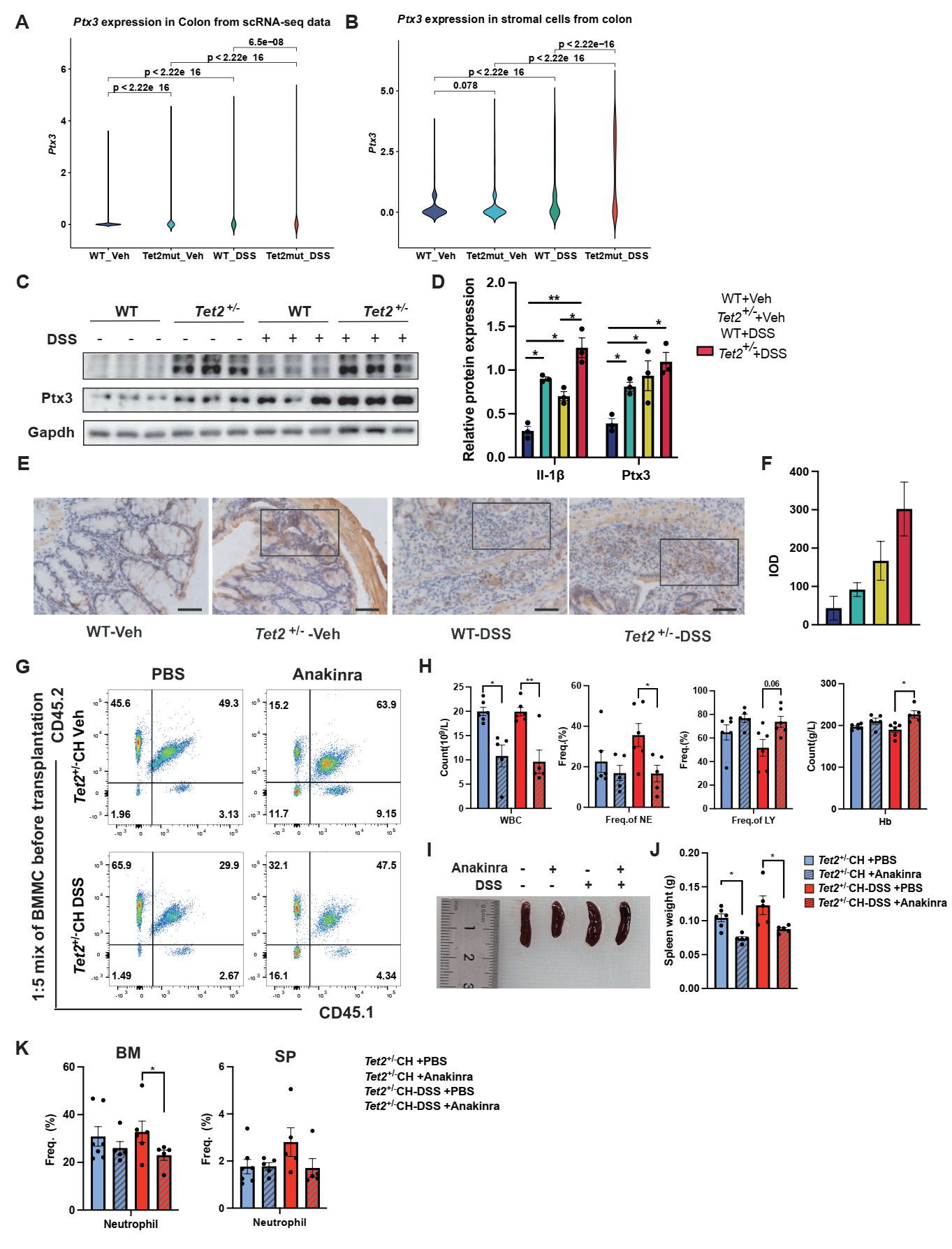
**

**Figure S10 The expression of Ptx3 and Il-1β was increased in the colon of *Tet2^+/-^* mice with chronic infection, related to Figure 6.**

(A-B) Expression of *Ptx3* in total colon tissues (A) and stromal cells (B) from the colon scRNA-seq dataset.

(C-D) The proteins level of Il-1β and Ptx3 were measured in colon tissues and quantified.

(E-F) Representative immunohistochemical (IHC) staining of Ptx3 in colon (E) and semi-quantitative analysis of immunohistochemistry staining of Ptx3 based on integrated optical density (IOD) (F). scale bar is 100 μm.

(G) Representative flow profiles of PB from the chimeric mice treated with Anakinra or PBS.

(H) Hematological parameters of PB were monitored at the end-point of the Anakinra treatment.

(I-J) Photography (I) and quantification of spleens (J) for the TedCH chimeric mice treated with DSS and/or Anakinra.

(K) Quantification of mature cells including neutrophils in bone marrow and spleen. BM, bone marrow; SP, spleen; PB, peripheral blood.

Data are shown as means ± SEMs. Number of biological repeats (animals): n = 5~7. Experiments of Anakinra treatment were repeated twice. *, p < 0.05; **, p < 0.01.
